# Health-related quality of life and overall survival: a prospective study in patients with head and neck cancer treated with radiotherapy

**DOI:** 10.1007/s11136-020-02716-x

**Published:** 2020-12-08

**Authors:** A. J. van Nieuwenhuizen, L. M. Buffart, J. A. Langendijk, M. R. Vergeer, J. Voortman, C. R. Leemans, I. M. Verdonck-de Leeuw

**Affiliations:** 1 Amsterdam University Medical Centers, Department of Otolaryngology-Head and Neck Surgery, Amsterdam, The Netherlands; 2 Amsterdam University Medical Centers, Vrije Universiteit Amsterdam, department of Epidemiology and Biostatistics, Amsterdam Public Health, Amsterdam, The Netherlands; 3 Amsterdam University Medical Centers, Vrije Universiteit Amsterdam, department of Medical Oncology, Cancer Center Amsterdam, Amsterdam, The Netherlands; 4 University Medical Center Groningen, Department of Radiation Oncology, University of Groningen, Groningen, The Netherlands; 5 Amsterdam University Medical Centers, Department of Radiation Oncology, Amsterdam, The Netherlands; 6 Amsterdam University Medical Centers, Department of Medical Oncology, Amsterdam, The Netherlands

**Keywords:** Head and neck cancer, Health-related quality of life, Survival

## Abstract

**Purpose:**

We aimed to examine whether pre-treatment, post-treatment and change in health-related quality of Life (HRQoL) is associated with survival, in patients with head and neck cancer (HNC).

**Methods:**

We included 948 newly diagnosed HNC patients treated with primary or adjuvant (chemo)radiotherapy with curative intent. The EORTC QLQ-C30 questionnaire was assessed pre-treatment and at 6 weeks, 6 months and 12 months post-treatment. Multivariable Cox regression analyses were performed to examine whether HRQoL at all time points and changes in HRQoL over time were associated with survival, after adjusting for demographic, clinical and lifestyle-related variables.

**Results:**

Higher HRQoL scores were significantly associated with improved 5-year overall survival at all time points, except for the subscale global QoL at 6 weeks. Changes in HRQoL at 6 weeks post-treatment compared to pre-treatment were not significantly associated with survival. Changes in physical (HR: 0.88 95% CI: 0.82–0.96) and emotional functioning (HR: 0.90 95% CI: 0.85–0.96) from pre-treatment to 6 months post-treatment and changes in global QOL, and physical, emotional, and social functioning from pre-treatment to 12 months post-treatment were significantly associated with survival.

**Conclusion:**

Higher HRQoL reported pre-treatment and post-treatment (6 weeks, 6 months and 12 months) are significantly associated with improved survival, as well as changes in HRQoL at 6 and 12 months compared to pre-treatment. Our results highlight the value of monitoring HRQoL and to identify those patients that report decreased or deteriorated HRQOL. This may help to further improve cancer care in a timely and efficient manner.

## Introduction

Many patients with head and neck cancer (HNC) have to deal with severe physical and psychosocial problems because of the disease and its treatment. Additionally, they are often confronted with HNC-specific problems, such as oral dysfunction, swallowing and speech impediments [1–10]. These disorders have a distinct impact on the health-related quality of life (HRQoL) of patients with HNC. It has been shown that the initial course of HRQoL during the first 2 years following treatment is favourable in HNC survivors compared to patients who ultimately succumb to the disease [9]. Furthermore, previous observational studies showed a significant association between HRQoL and survival, independently from other demographic, lifestyle-related and clinical factors [11–20]. In a previous systematic review, we found evidence for a significant association between pre-treatment physical functioning and survival, and between change in global QoL from pre-treatment to 6-month follow-up and survival in patients with HNC [21]. However, we noticed that only a small majority (58%) of the existing studies was of high quality. Particularly, 63% of the studies included in that review did not consider relevant confounders (e.g. 11 studies did not assess comorbidity, and seven studies did not assess smoking and alcohol consumption) [21].

As a consequence, it remains difficult to draw firm conclusions on the association between HRQoL and survival. Therefore, the aim of this prospective study was to examine whether pre-treatment HRQoL, HRQoL at 6 weeks, and 6 and 12 months after treatment and change in HRQoL is associated with survival, after adjusting for demographic, clinical, and lifestyle-related factors in patients with HNC.

## Patients and methods

### Study population

Between January 1999 and October 2009, all newly diagnosed patients with HNC who were planned to be treated with primary or adjuvant (chemo)radiotherapy in the Amsterdam University Medical Centres, location VUmc, completed questionnaires on HRQoL before treatment, and at 6 weeks, and 6 and 12 months after treatment as part of clinical routine. Patients were eligible for the current analyses if they: (1) were diagnosed with primary squamous cell carcinomas of the mucosal surfaces of the oral cavity, oropharynx, hypopharynx and larynx, (2) were treated with (chemo)radiotherapy or surgery combined with (chemo)radiotherapy with curative intent, (3) were ≥ 18 years old, (4) were able to read and understand the Dutch language and (5) completed the pre-treatment questionnaire. Patients were excluded if they had a distant metastasis, were previously treated with surgery or radiotherapy in the head and neck area, or brachytherapy, or had a serious cognitive impairment at baseline.

### Health-related quality of life

HRQoL was assessed using the 30-item European Organization for Research and Treatment of Cancer, (EORTC) Quality of Life Questionnaire core module (QLQ-C30) [22]. For the current analyses, we included the global quality of life (QoL) scale and the five function scales (physical, role, emotional, cognitive, and social functioning). Higher scores on the global QoL and functioning scales represent higher HRQoL.

#### Survival

Five-year survival was assessed by linking medical records to the Dutch death certificate register of the government, accessible for organizations with a public or societal task, such as hospitals. Survival was calculated from the date of inclusion (pre-treatment questionnaire) until death.

#### Demographic, lifestyle-related and clinical factors

Demographic (i.e. gender, age, socio-economic status (SES)), lifestyle-related (i.e. smoking in pack years, smoking history, alcohol use (units per day), alcohol abuse (≥5 units per day)), and clinical factors (i.e. tumour site, stage, human papillomavirus (HPV) status, types of treatment and comorbidity) were obtained from medical records. Socio-economic status was determined using zip codes of patients’ living area. Zip codes were translated to SES according to The Netherlands Institute for Social Research [23]. This system describes the social status of a district compared to other districts in The Netherlands using an algorithm based on mean income, percentage of people with low income, percentage of people with low education and percentage of people without a job. Therefore, the mean score of all districts in The Netherlands is zero. We dichotomized SES scores to high (> mean value) versus low (≤ mean value).

Tumour stage was determined according to the American Joint Committee on cancer (AJCC) TNM staging system (seventh ed., 2010). Tumour site was categorized into cancer of the oral cavity, HPV-positive oropharynx, HPV-negative oropharynx, larynx or hypopharynx. All biopsies of patients with oropharyngeal cancer were tested for HPV on formalin-fixed, paraffin-embedded tumour specimen according to a validated test algorithm [24, 25].

Treatment modality was categorized into radiotherapy alone, chemoradiation, or surgery followed by adjuvant (chemo)radiation. Additionally, we recorded whether the patients were treated with 3D-CRT (3-Dimensional Conformal Radiotherapy) or Intensity Modulated Radiotherapy (IMRT), which was introduced in our hospital in 2004. Comorbidity was assessed by a research physician (AvN) using the Adult Comorbidity Evaluation 27 (ACE-27) score [26], a validated chart built instrument examining the presence of any of the following medical conditions: cardiovascular, respiratory, gastro-intestinal, renal, endocrine, neurological, immunological, previous malignancies, psychiatric disorders, alcohol use, and severe overweight, resulting in a total comorbidity score of none, mild, moderate or severe.

### Statistical analysis

Descriptive statistics (mean, standard deviation (SD), or numbers and percentages) were generated for demographic, lifestyle-related, clinical factors, and HRQoL.

Univariable and multivariable Cox proportional hazard regression analyses were used to examine the association between HRQoL and survival. In the multivariable analyses, we adjusted for relevant demographic, lifestyle-related and clinical variables.

Separate models were built for each HRQoL subscale and for the different time points (pre-treatment, 6 weeks, 6 months and 12 months after treatment, and change in HRQoL at 6 weeks compared to pre-treatment, change at 6 months compared to pre-treatment, and change at 12 months compared to pre-treatment). In the regression analyses, we divided all HRQoL scores by 10 because such changes are considered clinically meaningful [27]. For all statistical analyses, *p* < 0.05 was considered statistically significant.

## Results

### Patient characteristics

From January 1999 and October 2009, 948 newly diagnosed patients with HNC met the inclusion criteria for the current analyses. All patients completed the questionnaire pre-treatment. After treatment, questionnaires were completed by 703 patients of the 947 alive (74%) at 6 weeks, 654 patients of the 914 alive (72%) at 6 months and 579 patients of the 838 alive (69%) at 12 months.

Demographic, lifestyle-related and clinical characteristics of the study population are presented in Table 1. The most frequent tumour site was larynx (43%). Among the patients with oropharyngeal cancer, 58% were diagnosed with a HPV-negative tumour (HPV status was unknown in 14%). Overall, 60% of patients were alive after 5 years.

**Table 1:** Pre-treatment demographic, lifestyle-related and clinical characteristics of the study population

Characteristics	Patients (*n* = 948)
Demographic factors	
Gender, *n* (%) male	692 (73%)
Age, mean (SD) years	62 (11)
High SES (above average), *n* (%)	139 (15%)
*Lifestyle-related factors*	
Smoking (pack years), mean (SD)	31 (22)
Former or current smoker, *n* (%)	806 (85)
Alcohol use (units per day), mean (SD)	3 (3)
Former or current alcohol abuse†, *n* (%)	262 (28)
*Clinical factors*	
Tumour site, *n* (%)	
Oral Cavity	152 (16)
Oropharynx	306 (32)
Oropharynx HPV positive*	86 (28)
Oropharynx HPV negative*	176 (58)
Oropharynx HPV unknown*	44 (14)
Larynx	413 (44)
Hypopharynx	77 (8)
Disease Stage, *n* (%)	
I	171 (18)
II	193 (20)
III	181 (19)
IV	402 (43)
Comorbidity, *n* (%)	
None	297 (31)
Mild	322 (34)
Moderate	239 (25)
Severe	90 (10)
Type of treatment, *n* (%)	
Radiotherapy	522 (55)
Chemoradiation	224 (24)
Primary surgery with adjuvant treatment	203 (21)
RT technique, *n* (%)	
IMRT	593 (63)
5-year overall survival rate (%)	570 (60)
Drop-out due to death, *n* (%)	
6 weeks	10 (1)
6 months	34 (4)
12 months	110 (12)

*n* number, *RT* radiotherapy, *SD* standard deviation, *SES* socio-economic status

†Alcohol abuse defined as ≥5 units of alcohol per day

*Numbers and percentages of total oropharyngeal cancer sites

### Health-related quality of life in relation to survival

Mean (SD) scores on the HRQoL subscales and results of Cox regression analyses are presented in Table 2. Adjusted for all included demographic, lifestyle-related and clinical factors, higher (better) scores on all subscales (global QoL, physical functioning, role functioning, emotional functioning, cognitive functioning and social functioning) as measured pre-treatment and at 6 and 12 months after treatment were significantly associated with longer survival (Table 2). At 6 weeks after treatment, higher scores on all subscales were also significantly associated with longer survival, except for global QoL.

**Table 2:** HRQoL scores and uni- and multivariable Cox regression analyses on the association between HRQoL and survival

	Mean (SD)	Univariable model HR (95% CI)	*p* value*	Multivariable model HR (95% CI) †	p value*
EORTC QLQ-C30 pre-treatment (*n* = 948)					
Global quality of life	66.6 (22.3)	0.89 (0.85–0.93)	0.00	0.91 (0.87–0.96)	0.00
Physical function	82.3 (20.8)	0.84 (0.81–0.88)	0.00	0.87 (0.83–0.91)	0.00
Role functioning	73.4 (32.3)	0.92 (0.90–0.95)	0.00	0.93 (0.90–0.96)	0.00
Emotional functioning	68.3 (23.4)	0.93 (0.90–0.97)	0.00	0.94 (0.90–0.97)	0.01
Cognitive functioning	85.1 (20.9)	0.92 (0.88–0.96)	0.00	0.91 (0.87–0.95)	0.00
Social functioning	82.4 (24.6)	0.92 (0.88–0.95)	0.00	0.91 (0.87–0.95)	0.00
EORTC QLQ-C30 6 weeks (*n* = 703)					
Global quality of life	66.2 (21.5)	0.90 (0.85–0.95)	0.00	0.94 (0.89–1.00)	0.06
Physical function	74.6 (22.3)	0.86 (0.82–0.90)	0.00	0.90 (0.85–0.95)	0.00
Role functioning	66.5 (30.6)	0.91 (0.88–0.95)	0.00	0.93 (0.90–0.97)	0.00
Emotional functioning	76.2 (23.6)	0.93 (0.89–0.98)	0.00	0.94 (0.89–0.99)	0.02
Cognitive functioning	83.1 (21.1)	0.92 (0.87–0.97)	0.00	0.93 (0.88–0.91)	0.01
Social functioning	77.6 (25.1)	0.93 (0.89–0.97)	0.00	0.93 (0.88–0.98)	0.00
EORTC QLQ-C30 6 months (*n* = 654)					
Global quality of life	71.0 (21.7)	0.86 (0.81–0.91)	0.00	0.87 (0.82–0.93)	0.00
Physical function	79.7 (19.9)	0.79 (0.75–0.84)	0.00	0.80 (0.75–0.86)	0.00
Role functioning	73.9 (29.2)	0.89 (0.85–0.93)	0.00	0.90 (0.86–0.94)	0.00
Emotional functioning	78.9 (24.1)	0.88 (0.84–0.92)	0.00	0.88 (0.83–0.93)	0.00
Cognitive functioning	85.2 (21.1)	0.91 (0.86–0.96)	0.00	0.89 (0.84–0.95)	0.00
Social functioning	82.5 (23.9)	0.89 (0.85–0.93)	0.00	0.89 (0.844–0.95)	0.00
EORTC QLQ-C30 12 months (*n* = 579)					
Global quality of life	73.9 (21.5)	0.82 (0.77–0.87)	0.00	0.81 (0.76–0.87)	0.00
Physical function	82.1 (19.5)	0.79 (0.74–0.85)	0.00	0.81 (0.74–0.87)	0.00
Role functioning	78.1 (28.1)	0.86 (0.82–0.91)	0.00	0.86 (0.81–0.91)	0.00
Emotional functioning	81.7 (22.2)	0.86 (0.81–0.92)	0.00	0.82 (0.76–0.88)	0.00
Cognitive functioning	86.2 (19.7)	0.90 (0.84–0.97)	0.00	0.89 (0.82–0.96)	0.00
Social functioning	85.3 (22.2)	0.85 (0.80–0.90)	0.00	0.84 (0.78–0.89)	0.00

†Adjusted for age, gender, socio-economic status, smoking (pack years), alcohol abuse (current or history), comorbidity, tumour site, tumour stage, treatment modality

*P of the log likelihood test

Higher global QoL and functioning scores indicate higher HRQoL (scale 0–100)

Table 3 presents the mean changes in HRQoL at 6 weeks, 6 months and 12 months after treatment, respectively, compared to pre-treatment. Changes in HRQoL from pre-treatment to 6 weeks after treatment were not significantly associated with survival for any of the subscales (Table 4). Deterioration in physical and emotional functioning at 6 months post-treatment compared to pre-treatment was significantly associated with shorter survival. Deterioration in global QoL, physical, emotional and social functioning at 12 months after treatment compared to pre-treatment was significantly associated with shorter survival.

**Table 3:** Mean (SD) change scores in HRQoL

	Mean (SD) change
EORTC QLQ-C30 Δ 6 weeks (*n* = 703)	
Global quality of life	−1.4 (23.7)
Physical function	−8.3 (20.8)
Role functioning	−7.2 (37.4)
Emotional functioning	6.9 (24.1)
Cognitive functioning	−2.2 (23.6)
Social functioning	−5.0 (28.1)
EORTC QLQ-C30 Δ 6 months (*n* = 654)	
Global quality of life	3.3 (23.6)
Physical function	−4.2 (18.8)
Role functioning	−1.0 (33.9)
Emotional functioning	9.9 (24.6)
Cognitive functioning	−0.6 (22.1)
Social functioning	−1.0 (28.3)
EORTC QLQ-C30 Δ 12 months (*n* = 579)	
Global quality of life	5.3 (23.5)
Physical function	−2.8 (18.9)
Role functioning	3.6 (34.9)
Emotional functioning	12.2 (23.0)
Cognitive functioning	−0.1 (20.9)
Social functioning	1.5 (27.1)

Δ change compared to pre-treatment. A negative mean change score indicates worsening of HRQoL after treatment compared to pre-treatment

**Table 4:** Uni- and multivariable Cox regression analyses on the association between changes in HRQoL after treatment compared to pre-treatment and survival

	Univariable model HR (95% CI)	p value*	Multivariable model HR (95% CI) †	p value*
EORTC QLQ-C30 Δ 6 weeks (*n* = 703)				
Global quality of life	1.01 (0.96–1.07)	0.62	1.02 (0.96–1.07)	0.59
Physical function	0.98 (0.93–1.04)	0.57	0.98 (0.93–1.04)	0.56
Role functioning	0.99 (0.96–1.02)	0.61	0.99 (0.96–1.03)	0.71
Emotional functioning	0.99 (0.94–1.04)	0.74	1.00 (0.95–1.05)	0.86
Cognitive functioning	1.01 (0.96–1.06)	0.81	1.01 (0.96–1.07)	0.66
Social functioning	1.00 (0.96–1.05)	0.89	1.00 (0.95–1.05)	0.96
EORTC QLQ-C30 Δ 6 months (*n* = 654)				
Global quality of life	0.95 (0.90–1.01)	0.10	0.94 (0.88–1.00)	0.05
Physical function	0.90 (0.84–0.97)	0.01	0.88 (0.82–0.96)	0.00
Role functioning	0.98 (0.94–1.02)	0.33	0.97 (0.93–1.02)	0.23
Emotional functioning	0.91 (0.86–0.97)	0.00	0.90 (0.85–0.96)	0.00
Cognitive functioning	0.96 (0.90–1.03)	0.25	0.96 (0.90–1.03)	0.24
Social functioning	0.96 (0.92–1.01)	0.12	0.97 (0.92–1.03)	0.29
EORTC QLQ-C30 Δ 12 months (*n* = 579)				
Global quality of life	0.93 (0.86–0.99)	0.03	0.90 (0.84–0.97)	0.00
Physical function	0.91 (0.83–0.99)	0.03	0.89 (0.81–0.97)	0.01
Role functioning	0.97 (0.92–1.02)	0.19	0.96 (0.91–1.01)	0.12
Emotional functioning	0.91 (0.84–0.97)	0.01	0.87 (0.81–0.94)	0.00
Cognitive functioning	0.97 (0.89–1.05)	0.45	0.96 (0.88–1.04)	0.33
Social functioning	0.92 (0.86–0.98)	0.01	0.90 (0.84–0.96)	0.00

†adjusted for age, gender, socio-economic status, smoking (pack years), alcohol abuse (current or history), comorbidity, tumour site, tumour stage, treatment

*p value of the log likelihood

Δ change compared to pre-treatment

## Discussion

This comprehensive study among a large group of patients with HNC showed that better HRQoL was significantly associated with longer survival, adjusted for demographic, lifestyle-related and clinical factors. This association was found for global QoL, and physical, role, emotional, cognitive, and social functioning before treatment as well as 6 weeks, 6 months and 12 months after treatment. Changes in HRQoL at 6 weeks after treatment compared to pre-treatment were not significantly associated with survival. However, deterioration in physical and emotional functioning at 6 and 12 months after treatment compared to pre-treatment was significantly associated with shorter survival, as well as deterioration in global QoL and social functioning at 12 months.

Our finding that worse HRQoL before and after treatment is significantly associated with shorter survival supports results from previous observational studies in patients with HNC [11–16, 18–20, 28]. In contrast to previous studies that reported an association with survival of some HRQoL domains and measured at different time points [21, 29, 30], we consistently found that global QoL and all function domains of HRQoL assessed at all time points during the first year after cancer diagnosis were associated with survival. The inconsistent findings across the different subscales and time points in the previous studies may be related to the smaller sample sizes in those studies [12, 14, 16, 17, 19, 20, 31, 32] and the heterogeneity of the tumour sites and stages [13, 15, 33–41].

Interestingly, where HRQoL measured 6 weeks after treatment was significantly associated with survival, change in HRQoL, as measured at 6 weeks after treatment compared to pre-treatment was not. This may be explained by the fact that shortly after treatment, many patients still suffer from the acute side effects of treatment and change in HRQoL at short term is not yet a discriminating factor [6, 9]. Most of these acute adverse effects are absent from 6 months onwards [1, 2, 6, 9].

Worse physical and emotional function at 6 and 12 months after treatment compared to pre-treatment was significantly associated with shorter survival. The association between physical functioning and survival has been shown in previous studies, also in patients with cancer types other than HNC [29, 30, 42]. For instance, a recent study in patients with advanced colorectal cancer revealed that physical functioning assessed with patient-reported outcomes had more prognostic value in predicting overall survival than physician-assessed world health organization (WHO) performance status [43].

The association between emotional functioning and survival corresponds with findings from a previous longitudinal study in a large cohort of patients HNC showing a significant association between depressive symptoms and shorter survival [44]. These findings support earlier studies that reported a significant association between (symptoms of) depression and survival in the community and disease-specific populations [45, 46].

In addition to deteriorations in physical and emotional functioning, deteriorations in global QOL and social functioning at 12 months after treatment were also associated with reduced survival. Perhaps, reduced physical and emotional functioning over time also affects global QOL and social functioning. Shortly after diagnoses these problems could be more thoroughly present in patients’ lives, where the effects on social or global QoL are postponed. However, when acute symptoms have stabilized after 12 months [1, 2, 6, 9] patients will be more aware of the persistent effects of HNC and its treatment and the consequences on their social life and global QoL. On the other hand, patients with advanced illness could also not be able to perform in social activities.

Based on our results, monitoring changes in HRQOL (especially physical and emotional functioning) over time in clinical practice seems important, as these scores may be sensitive for signalling clinical deterioration. Symptom monitoring (such as dyspnoea, fatigue and pain) in routine care of patients seems to be associated with increased survival compared to usual care [47]. This can be explained by the early responses of nurses to symptom alerts with clinical interventions, and better chemotherapy toleration compared to the usual care group [47].

Strengths of our study include the large sample of newly diagnosed patient with HNC, allowing to incorporate multiple relevant demographic, lifestyle-related and clinical factors in our statistical models, including HPV status. Another strength is that we investigated the association between survival and HRQoL at different time points before and after treatment. However, some limitations must be noted. We included only patients that received primary or adjuvant (chemo)radiotherapy, and thus excluded patients treated with surgery alone. Also, the baseline HRQOL in this group was performed after surgery, before postoperative treatment began. Radical surgery for locally advanced HNC is typically quite morbid, and this may have negatively influenced baseline HRQOL scores in this study. Furthermore, the study cohort was treated before 2010, thus not including patients who were treated by recent improvements in (chemo)radiotherapy. These limitations may hamper generalizability of the results. Furthermore, because demographic, lifestyle-related, and clinical variables were retrieved from medical records, we may have missed other important variables that may be predictive for survival such as physical activity, nutritional intake, or marital status, income and occupation [48], and possibly other (head and neck) cancer symptoms. Finally, we were unable to retrieve data on disease-specific survival, which limited our analysis to overall survival.

In conclusion, (change in) HRQoL is significantly associated with survival in addition to demographical, lifestyle-related and clinical measures, not only pre-treatment, but also 6 weeks, 6 months and 12 months after treatment. This highlights the value of monitoring HRQoL in (clinical) practice to identify those patients that report changes in HRQOL at 6 and 12 months after treatment. This may help to further improve cancer care in a timely and efficient manner.

## Data Availability

Data are available upon request.

## Abbreviations

EORTC QLQ-C30: European Organization for Research and Treatment of Cancer Quality of Life Questionnaire core module
HNC: head and neck cancer
HPV: human papillomavirus
HRQoL: health-related quality of life
SES: socio-economic status
3D-CRT: 3-Dimensional Conformal Radiotherapy
IMRT: Intensity Modulated Radiotherapy
ACE-27: Adult Comorbidity Evaluation 27
SD: standard deviation
